# Defined Essential 8™ Medium and Vitronectin Efficiently Support Scalable Xeno-Free Expansion of Human Induced Pluripotent Stem Cells in Stirred Microcarrier Culture Systems

**DOI:** 10.1371/journal.pone.0151264

**Published:** 2016-03-21

**Authors:** Sara M. Badenes, Tiago G. Fernandes, Cláudia S. M. Cordeiro, Shayne Boucher, David Kuninger, Mohan C. Vemuri, Maria Margarida Diogo, Joaquim M. S. Cabral

**Affiliations:** 1 Department of Bioengineering, and Institute for Bioengineering and Biosciences, Instituto Superior Técnico, Universidade de Lisboa, Lisboa, Portugal; 2 Thermo Fisher Scientific, Cell Biology, Life Sciences Solutions, Frederick, Maryland, United States of America; Cornell University, UNITED STATES

## Abstract

Human induced pluripotent stem (hiPS) cell culture using Essential 8™ xeno-free medium and the defined xeno-free matrix vitronectin was successfully implemented under adherent conditions. This matrix was able to support hiPS cell expansion either in coated plates or on polystyrene-coated microcarriers, while maintaining hiPS cell functionality and pluripotency. Importantly, scale-up of the microcarrier-based system was accomplished using a 50 mL spinner flask, under dynamic conditions. A three-level factorial design experiment was performed to identify optimal conditions in terms of a) initial cell density b) agitation speed, and c) to maximize cell yield in spinner flask cultures. A maximum cell yield of 3.5 is achieved by inoculating 55,000 cells/cm^2^ of microcarrier surface area and using 44 rpm, which generates a cell density of 1.4x10^6^ cells/mL after 10 days of culture. After dynamic culture, hiPS cells maintained their typical morphology upon re-plating, exhibited pluripotency-associated marker expression as well as tri-lineage differentiation capability, which was verified by inducing their spontaneous differentiation through embryoid body formation, and subsequent downstream differentiation to specific lineages such as neural and cardiac fates was successfully accomplished. In conclusion, a scalable, robust and cost-effective xeno-free culture system was successfully developed and implemented for the scale-up production of hiPS cells.

## Introduction

Human induced pluripotent stem (hiPS) cells are capable of self renewing indefinitely, and to differentiate into all the cell types of the human body [1]. Because of these characteristics, analogous to human embryonic stem (hES) cells, hiPS cells are promising sources for several biomedical applications [2]. However, to fully realize the potential of hiPS cells for cellular therapy, drug screening and disease modelling, the development of standardized and robust scalable processes to produce large numbers of these cells while maintaining their critical biological functionality and safety are of prime importance.

Typically, hiPS cells are expanded using adherent static cell culture systems that cannot provide a sufficient number of cells for downstream applications, presenting low cell yields and inherent variability of the culture process and of the final product. Translating cell culture from static plates to suspension systems is needed to achieve scalability of the process. Stirred bioreactors are an appropriate culture system for moderate large-scale cell production given their robustly controlled operation and well-established scale-up protocols [3,4,5]. Several methodologies for human pluripotent stem (hPS) cell culture in these systems have been implemented in the last few years, including cultivation of cells encapsulated typically inside hydrogels [6,7], adherent onto microcarriers [8,9], or as 3D aggregates in suspension [10,11]. Microcarrier technology confers distinct advantages as it provides homogeneous culture conditions to the cells, large surface areas for cell adhesion and growth [12,13] and importantly, a large surface/volume ratio. Also, microcarrier culture on fully controlled bioreactors allows monitoring and controlling of environmental parameters, and can be scaled up relatively easily. Nevertheless, despite recent progress on scalable microcarrier hPS cell suspension culture, most of the methods are based on the use of non-defined extracellular matrix (ECM) extracts, such as Matrigel™ or Geltrex™, as surface for cell adherence on microcarriers [14,15,16], and commercially available serum-free media, such as mTeSR™ and StemPro^®^ [14,17,18], that contain animal-derived products.

Envisioning the bioprocess translation to Good Manufacturing Practice (GMP) standards, great efforts have been made towards the translation of scalable culture systems to chemically defined and xeno-free conditions. A completely defined medium, Essential 8™, that consists of only eight components, was recently developed [19,20,21], and several other studies have been reporting defined surfaces that support long-term hiPS cell culture, like vitronectin, laminin, fibronectin and various synthetic peptides [15,18,22,23]. Nevertheless, the use of Essential 8™ medium to support expansion of hiPS cells on microcarriers coated with defined substrates has never been reported.

To design a bioprocess to produce a biomedical product, it is of foremost importance to set up robust and reproducible production practices. Therefore, robust predictive strategies to evaluate process parameters that will impact culture output need to be developed. Rational design of experiments can provide a model to predict the culture output as a function of multiple culture parameters [24,25]. Therefore, in this work, we implemented a stirred culture system based on the use of vitronectin-coated microcarriers and Essential 8™ medium for the scalable expansion of hiPS cells, using 50 mL spinner flasks. Importantly, a three-level factorial design model was used to identify the optimal conditions that maximize cell yield. Finally, given the potential applications of hiPS cells in differentiation and lineage specification studies, we investigated the differentiation capacity of hiPS cells cultured on microcarriers, under xeno-free chemically defined conditions, to cardiomyocytes and to neural progenitor cells.

## Materials and Methods

### Cells and microcarriers

Gibco™ human induced pluripotent stem cell line used in this work was derived from CD34+ cells of healthy donors (Life Technologies). The hiPS cells were routinely cultured on Geltrex (1:60, Life Technologies) -coated 6-well plates in Essential 8 (E8) medium (Life Technologies), in a humidified 5% CO_2_ incubator at 37°C. The medium was refreshed daily and cells were routinely passaged at a split ratio of 1:4 using the EDTA method [26], when colonies reached 80% confluence. hiPS cells were adapted to Vitronectin (rhVTN-N, Life Technologies)–coated plates for two passages prior to inoculation onto microcarriers. Cells were routinely evaluated for karyotype abnormalities by conventional cytogenetics using the services of Genomed SA (Lisbon, Portugal).

Polystyrene microcarriers (Solohill Engineering, Inc.), with 360 cm^2^/g of superficial area, were used to support cell growth. Microcarriers were mixed during 1 h with Ethanol 70% (Sigma) at room temperature and washed 3 times with sterile phosphate-buffered saline (PBS). Coating of microcarriers was performed for 2 h at room temperature with Vitronectin in sterile PBS, using 0.5 μg/cm^2^. Geltrex-coated microcarriers were used (coating: 0.25 mL/cm^2^ of Geltrex solution (1:60)) as a control. Prior to cell inoculation, microcarriers were incubated (at 37°C) for 30 min in culture medium.

### Inoculation of hiPS cells on microcarriers

#### Inoculation as single cells

The protocol for the inoculation of hiPS cells on microcarriers as single cells was described recently [27].

#### Inoculation as clumps

Cells were incubated for 5 min with Cell Dissociation Buffer (Life Technologies) at room temperature, using the EDTA method [26]. Cells were then collected and inoculated on microcarriers with or without ROCK inhibitor (10 μM, Y27632, from StemGent) for the first 24 h of culture.

### hiPS cell expansion on microcarriers

#### Static culture

The protocol for the screening of microcarriers for hiPS cell expansion under static culture in low-attachment 24-well plates (Corning Inc.) was recently published [27]. We used 3 cm^2^ of microcarrier superficial area per well and cells were inoculated at an initial density of 5x10^4^ cells/cm^2^. Geltrex- and vitronectin-coated polystyrene microcarriers (GM and VtnM) were tested. 80% of E8 medium was changed daily for 5 days. The cell yield in total cell number was calculated as the ratio *X*_*day5*_*/X*_*i*_, where *X*_*day5*_ is the number of viable cells, attached to the microcarriers, at day 5, and *X*_*i*_ is the number of cells inoculated at day 0.

#### Spinner flask culture

The expansion of hiPS cells in a microcarrier stirred suspension culture was performed in presiliconized (Sigmacote, Sigma) spinner flasks (StemSpan^TM^, StemCell Technologies), with a working volume of 50 mL. The impeller was composed of a horizontal magnetic stir bar with a vertical paddle. Agitation was obtained by a magnetic stirrer platform (Variomag, Biosystem), which was placed inside a 5% CO_2_ incubator at 37°C.

Cells were seeded as small clumps, at an initial density of 3, 5 or 7x10^4^ cells/cm^2^, using a total of 1 g of coated polystyrene microcarriers (360 cm^2^/spinner) in 25 mL of E8 medium under static conditions, to promote cell-microcarrier contact. Medium was supplemented with ROCK inhibitor (10 μM) for the first 24 h after inoculation. After 24 h, the medium was replaced and adjusted to 50 mL of fresh E8 medium. Subsequently, an intermittent stirring (3 min at 40 rpm every 2 h) was performed overnight to promote cell-cell and cell-microcarrier contact. Thereafter, the culture was continuously stirred at 30, 50 or 70 rpm and feeding was performed on a daily basis by replacing 80% of volume with fresh pre-warmed medium.

For spinner flask cultures, cell attachment efficiency to the microcarriers was calculated as the percentage of *X*_*day1*_*/X*_*i*_, where *X*_*day1*_ is the number of the viable cells attached to the microcarriers at day 1 of the culture and *X*_*i*_ is the number of cells inoculated at day 0. The maximum cell yield was calculated as the ratio *X*_*max*_*/X*_*i*_, where *X*_*max*_ is the maximum cell number, attached to the microcarriers, achieved during the culture.

#### Sampling

Duplicate 700 μL samples of the culture were collected from the spinner flasks everyday. In order to detach the cells, microcarriers were incubated with 0.05% trypsin (Life Technologies) at 37°C for 10 min, in the heater mixer set at 750 rpm. After dissociation by pipetting, the mixture was filtered through a 100 μm mesh (cell strainer, from BD Biosciences) to remove the microcarriers. Cells were then centrifuged at 210 *g* for 5 min and viable and dead cells were determined by counting in a hemocytometer under optical microscope, using the trypan blue dye exclusion test.

#### Cell harvesting from the microcarriers and re-plating

The cell harvesting and re-planting protocol at the end of the culture (static or dynamic) was performed as previously described [27]. Cells were resuspended in E8 medium supplemented with ROCK inhibitor (10 μM) and then inoculated at a density of 5x10^4^ cells/cm^2^ of well area on GP.

### Experimental design

The effects of two independent variables, initial cell density and agitation rate, on the cell yield were determined using a face-centered composite design (FC-CD) approach using STATISTICA software (StatSoft, Tulsa, OK). Each independent variable was evaluated at three different coded levels (low (−1), central (0) and high (+1)) as portrayed in S1 Table and combined in a FC-CD design set up described as:

$$N=2^{k-p}+2k+C_{0}$$

where *N* is the number of experiments, *k* is the number of variables (*k* = 2), *p* the fractionalization number (in a full design, *p* = 0) and *C*_*0*_ is the number of central points, that provides estimation of the experimental error. Accordingly, a total of 12 [2^2−0^+ (2×2) + 4] independent experiments were performed.

The data was fitted to a full quadratic model (including linear and non-linear effects, plus two-way interaction) as follow:

$$Y=\beta_{0}+\beta_{1}X_{1}+\beta_{11}{X_{1}}^{2}+\beta_{2}X_{2}+\beta_{22}{X_{2}}^{2}+\beta_{12}X_{1}X_{2}$$

where *Y* is the response measured or dependent variable (cell yield), *X*_*1*_ and *X*_*2*_ are the two independent variables, *β*_*0*_ is the intersect; *β*_*1*_ and *β*_*2*_ are the linear main effects, *β*_*11*_ and *β*_*22*_ are the quadratic coefficients, and *β*_*12*_ is the coefficient for the second order interaction. The error of the prediction was estimated from the error obtained by the genuine replicates performed on the central points of the matrix, done at least in 4 independent experiments [28]. The coefficient of regression (R^2^) was also determined by the software.

### Characterization of hiPS cells and derivatives

#### Antibodies

Primary antibodies used for immunofluorescence microscopy and flow cytometry included OCT4 (1:750) and NANOG (1:5000) (Milipore), SOX2 (1:1000) (R&D Systems), SSEA4 (1:135), SSEA4-PE (1:10), TRA-1-60 (1:135), TRA-1-60-PE (1:10) and TRA-1-81 (1:135) (StemGent). Secondary antibodies included goat anti-mouse IgG Alexa Fluor– 488 or 546 (1:500 or 1:1000), goat anti-rabbit IgG Alexa Fluor 546 (1:1000)–Invitrogen; and isotypes used for control in flow cytometry tests included anti-mouse IgM-PE (Miltenyi Biotec) and anti-mouse IgG-PE (1:10) (StemGent). Immuncytochemistry against markers from the three germ layers was performed using antibodies against alpha smooth muscle actin (α-SMA; mouse: 1:1000; Dako), neuron-specific class III β-Tubulin (TUJ1; mouse: 1:20 000; Covance) and SOX17 (mouse: 1:1000; R&D Systems), for the mesoderm, ectoderm and endoderm, respectively. Cardiomyocyte marker was Troponin T cardiac isoform antibody (13–11) (cTNT; mouse: 1:500; Thermo Scientific). Neural progenitor cell markers were NESTIN (mouse: 1:1000; R&D Systems) and paired box gene 6 (PAX6; rabbit: 1:1000; Covance).

#### Flow cytometry

Cells were kept at 4°C in 2% (v/v) paraformaldehyde (PFA, from Sigma). For surface staining, approximately 5x10^5^ cells were resuspended in 100 μL of FACS buffer (3% (v/v) Fetal Bovine Serum (FBS, from Invitrogen) in PBS) with the diluted primary antibody, and incubated for 15 min at room temperature in the dark. Cells were washed twice with PBS and resuspended in 300 μL of PBS to be analysed by flow cytometry (FACSCalibur, Becton Dickinson). For negative controls, cells were incubated with the appropriate isotypes. For intracellular staining, the protocol used is described by Miranda et al. [29]. For the negative controls, cells were incubated only with 3% (v/v) Normal Goat Serum (NGS, from Sigma) in PBS. The CellQuest software (Becton Dickinson) was used for all acquisition/analyses.

#### Immunocytochemistry

For surface antigens, after removing the culture medium, cells were incubated for 30 min at 37°C in the presence of the primary antibodies diluted in medium. Cells were washed 3 times with PBS and incubated in the dark for 30 min, at 37°C, with the secondary antibodies. For intracellular staining, the protocol used is described by Miranda et al. [29]. Cells were examined using a fluorescence microscope (Leica DMI3000B/Nikon Digital Camera Dxm1200F).

#### RT-PCR

RNA was isolated using PureLink^®^ RNA Mini Kit (Life Technologies). cDNA was synthetized using 1 μg of total RNA and the High Capacity cDNA Reverse Transcriptase kit (Life Technologies). StepOne QST Real-Time Polymerase Chain Reaction (RT-PCR) was performed using the TaqMan™ Gene Expression Assay (Applied Biosystems) (S2 Table).

#### *In vitro* hiPS cell differentiation potential

hiPS cell differentiation potential was evaluated *in vitro* via embryoid body (EB) formation and spontaneous differentiation. Cells from a spinner flask culture were harvested and inoculated as single-cells in GP. At 80% confluence, cells were passaged with EDTA treatment to a 6-well low-attachment plate in EBs medium (DMEM with 20% (v/v) FBS, 1% (v/v) MEM-non essential amino acids, 1mM sodium pyruvate, 0.1 mM β–mercaptoethanol and 1% (v/v) Penicillin/Streptomycin, all from Invitrogen), supplement with ROCK inhibitor for the first 24 h. Medium was changed every 2 days for 4 weeks thereafter. EBs were then dissociated with trypsin 0.025% and cells inoculated in a 24-well plate coated with 4 μg/mL laminin (StemGent) and 10 μg/mL poly-D-lysine (Sigma). Medium was changed every 2 days for 1 week. Finally, cells were stained with anti-SOX17, TUJ1 and α-SMA antibodies.

#### Directed hiPS cell cardiomyocyte differentiation

The Gibco^®^ hPS cell Cardiomyocyte Differentiation Kit (Life Technologies) was used to induce cardiomyocyte differentiation of hiPS cells adherent to confluent microcarriers from a spinner flask culture (without cell harvesting). Confluent microcarriers were placed in a 24-well low-attachment plate (3 cm^2^ of microcarriers area/well) and the protocol was performed using manufacture’s instructions. Also, cells harvested from the microcarriers at the end of the spinner flask culture were inoculated as single-cells in GP, and at 80% confluence, cardiomyocyte differentiation was initiated. On both cases, at the end of the differentiation protocol, cells were stained for cTNT and OCT4 markers.

#### Neural induction by Dual-SMAD inhibition

Confluent microcarriers from a spinner flask culture were placed in 24-well low-attachment plate (3 cm^2^ of microcarriers area/well). N2B27 medium supplemented with 10 μM of SB431542 (SB, Sigma) and 100 nM of LDN193189 (LDN, StemGent) was added and replaced daily for 12 days. N2B27 medium is composed of a 1:1 mixture of Dulbecco’s modified Eagle´s medium (DMEM)/F12 and Neurobasal medium supplemented with 1x N2 and 1x B27 (Life Technologies). At the end of the differentiation protocol, cells attached to microcarriers were stained with NESTIN, PAX6 and OCT4 markers. Also, differentiated cells attached to the microcarriers were dissociated by pipetting and plated on GP in the same N2B27-based medium. This medium was daily replaced for 12 days. At day 12, cells were stained for NESTIN, PAX6 and OCT4 markers.

### Statistical analysis

All data presented show n = 3 replicates, unless stated otherwise. Error bars represent the standard error of the mean (SEM).

## Results

### Xeno-free surfaces for adherent hiPS cell culture in E8 medium

**Fig 1:**
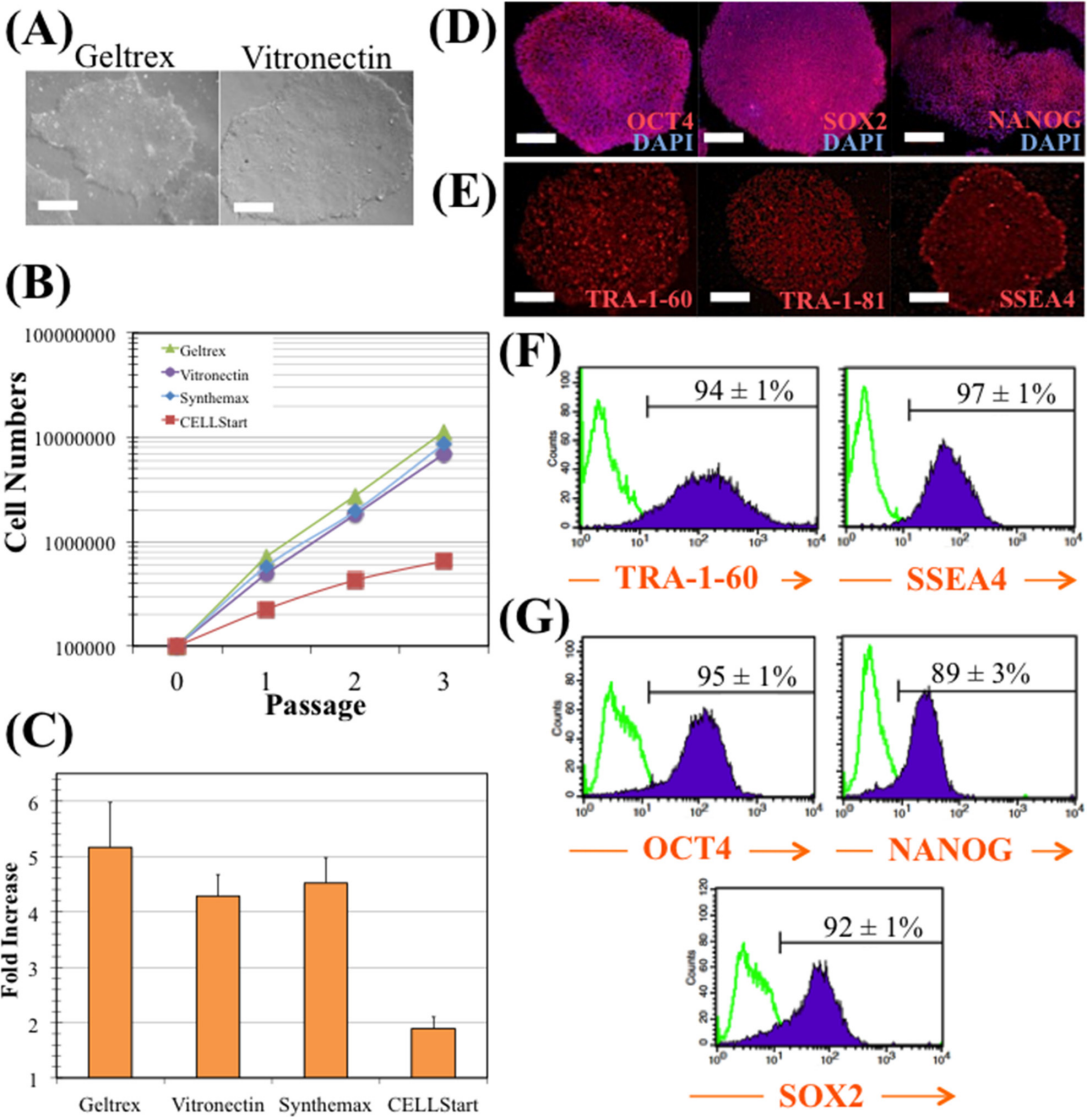
hiPS cell maintenance in E8 medium and vitronectin. (A) Colonies cultured with E8 medium in Geltrex (left panel), and in vitronectin (right panel). Scale bars—100μm. (B) Cumulative fold increase in total cell numbers over four passages with E8 medium in Geltrex (green), in vitronectin (purple), in Synthemax^®^ (blue) or in CELLStart™ (red). (C) Average fold increase per passage. The fold increase was 5.2 for cells growing in Geltrex, 4.3 for vitronectin, 4.5 for Synthemax^®^ and 1.9 for CELLStart™. Error bars represent SEM (standard error of the mean). Four passages and two independent experiments were performed. (D) Immunostaining of colonies cultured with E8 medium in vitronectin. Colonies were stained for pluripotency markers OCT4, SOX2, and NANOG, and nuclei counterstained with DAPI. Scale bars—100μm. (E) Immunostaining of colonies cultured with E8 medium in vitronectin. Colonies were stained for pluripotency surface markers TRA-1-60, TRA-1-81, and SSEA4. Scale bars—100μm. (F) and (G) Flow cytometry analysis of pluripotency surface markers (TRA-1-60 and SSEA4) (F) and transcription factors (OCT4, NANOG, SOX2) (G) in cells expanded with E8 medium and vitronectin.

In order to select the best xeno-free substrate for expansion of hiPS cells in combination with E8 culture medium, different substrates were tested and compared. Since the ability of vitronectin (Vtn) surfaces to support long-term hiPS cell expansion in xeno-free E8 medium has been described in the literature [19], the model hiPS cell line was seeded onto Vtn and Geltrex surfaces and cultured in E8 medium. As shown in Fig 1A, no significant differences were found in cell morphology between hiPS cells cultured on these two surfaces. On both cases, hiPS cells demonstrated a typical morphology of tightly packed colonies with defined borders and a high nucleus-to cytoplasm ratio.

Comparison studies were performed using different substrate surfaces for the adhesion, expansion and serial passaging of hiPS cells. Besides Geltrex and Vtn, CELLStart™ (Life Tecnhologies) and Synthemax^®^ (Corning Inc.) surfaces were also evaluated. As it can be seen in Fig 1B, cell growth kinetics were similar when hiPS cells were cultured on all surfaces, except on CELLStart™ surface. As shown in Fig 1C, cells cultured on Geltrex surface presented the highest fold increase (5.2±0.8), which could be expected due to its complex and rich protein composition [30]. However, this non-defined ECM extract surface may be a source of xenogeneic risk. In the same figure, it is shown a similar cell fold expansion when culturing the cells on Vtn (4.3±0.4) and Synthemax^®^ (4.5±0.5) surfaces. Although Synthemax^®^ is a chemically-synthesized substrate [31], Vtn is more cost-effective adhesion-promoting reagent [22] as was evaluated in the literature [30]. hiPS cells were then cultured during four consecutive passages on Vtn surface and immunofluorescence microscopy was performed to evaluate the expression of the intracellular and extracellular pluripotency markers OCT4, SOX2 and NANOG (with the corresponding DAPI stains of the nuclei), and TRA-1-60, TRA-1-81 and SSEA4, respectively (Fig 1D and 1E). The fluorescence images indicated that hiPS cells can be maintained in their undifferentiated state on Vtn surface. Moreover, flow cytometry analysis revealed consistently high expression levels of pluripotent markers TRA-1-60 (94±1%), SSEA4 (97±1%), OCT4 (95±1%), NANOG (89±3%) and SOX2 (92±1%) (Fig 1F and 1G). Finally, it was also verified that hiPS cells consistently displayed a normal karyotype (46 XX) after four passages on Vtn-coated tissue culture plates, in E8 medium (data not shown). In conclusion, the combination of Vtn surfaces and E8 medium support robust and long-term culture of undifferentiated hiPS cells under adherent static conditions.

### hiPS cell expansion on vitronectin-coated microcarriers: inoculation strategy

After demonstrating that Vtn could support the long-term culture of hiPS cells in xeno-free E8 medium, this matrix was used to coat polystyrene microcarriers and then to implement a scalable culture. Vtn-coated microcarriers were inoculated with 5x10^4^ cells/cm^2^ in low attachment 24-well plates.

**Fig 2:**
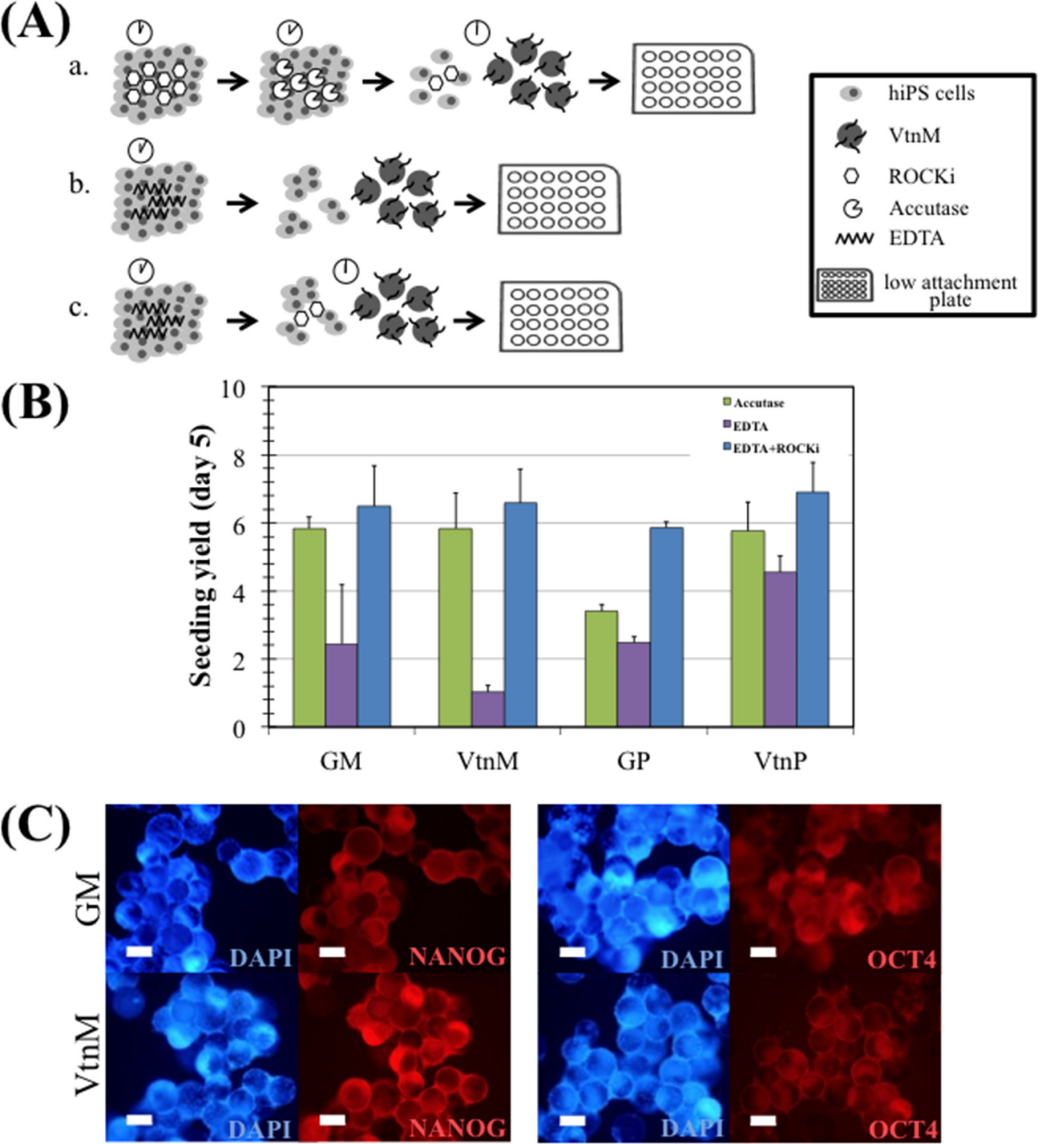
hiPS cell expansion on microcarriers in E8 medium, under static conditions. (A) Three different inoculation strategies: a. accutase, b. EDTA and c. EDTA+ROCKi were compared. (B) Seeding yield after 5 days of culture of hiPS cells on Geltrex coated polystyrene microcarriers (GM) and plate (GP), vitronectin coated polystyrene microcarriers (VtnM) and plate (VtnP), using the three different inoculation strategies: accutase (green), EDTA (purple) and EDTA+ROCKi (blue). Cells were inoculated at a 5x10^4^ cells/cm^2^ density. Error bars represent SEM (standard error of the mean). Three replicate wells were performed for each condition. (C) Immunostaining of hiPS cells cultured on microcarriers with E8 medium at day 5. Cells were stained for pluripotency markers NANOG (left panel) and OCT4 (right panel), and nuclei counterstained with DAPI. Scale bars– 100 μm.

Three inoculation strategies were evaluated (Fig 2A). In strategy (a) cells were incubated for 1h with ROCK inhibitor, dissociated with Accutase and inoculated as single cells in the presence of ROCK inhibitor for 24 h; in strategy (b), cells were dissociated with EDTA treatment and inoculated as cell clumps; and in strategy (c) cells were dissociated with EDTA treatment and were inoculated as cell clumps, in the presence of ROCK inhibitor for 24 h.

When hiPS cells were treated with EDTA for 3 minutes, this resulted in the formation of small clumps that were able to survive. However, due to the considerable size of these clumps (8–12 cells) cells tend to grow as aggregates rather than attaching onto microcarriers. Consequently, the time of incubation with EDTA was increased to 5 min in order to obtain smaller clumps (3–6 cells) and allow cell adhesion to the microcarriers. Since higher incubation time with EDTA had to be performed in the microcarrier-based culture, the addition of ROCK inhibitor for the first 24 h of culture was considered beneficial for smaller clump survival.

In Fig 2B, it can be observed the cell yield (see Materials and Methods) for the three different inoculation strategies, after 5 days of static culture of hiPS cells on Vtn-coated polystyrene microcarriers (VtnM) and plate (VtnP). Cell expansion on Geltrex-coated polystyrene microcarriers (GM) and plate (GP) were evaluated as a control. The highest cell yields were obtained using the inoculation strategy (c), and results with VtnM (5.8±1.0 for strategy (a), 1.0±0.2 for strategy (b) and 6.6±1.0 for strategy (c)) were similar to the ones obtained with GM. Moreover, for strategy (c), the culture on microcarriers showed similar yields to the static culture on plate (6.4±0.7). The inclusion of the small molecule Y27632 (ROCK inhibitor) has already been reported [19,32] to improve initial cell survival and to support high clonal efficiency. Therefore, cell-microcarrier adhesion efficiency was improved and higher cell yields were obtained in this case. Importantly, as presented in Fig 2C, after 5 days of culture, hiPS cells cultured on VtnM and GM, stained positively for NANOG and OCT4 pluripotency intracellular markers. Considering these results, strategy (c) was chosen for scaling-up the microcarrier-based culture.

### Optimization of hiPS cells expansion on a scalable stirred spinner flask culture by a face-centered composite design

**Fig 3:**
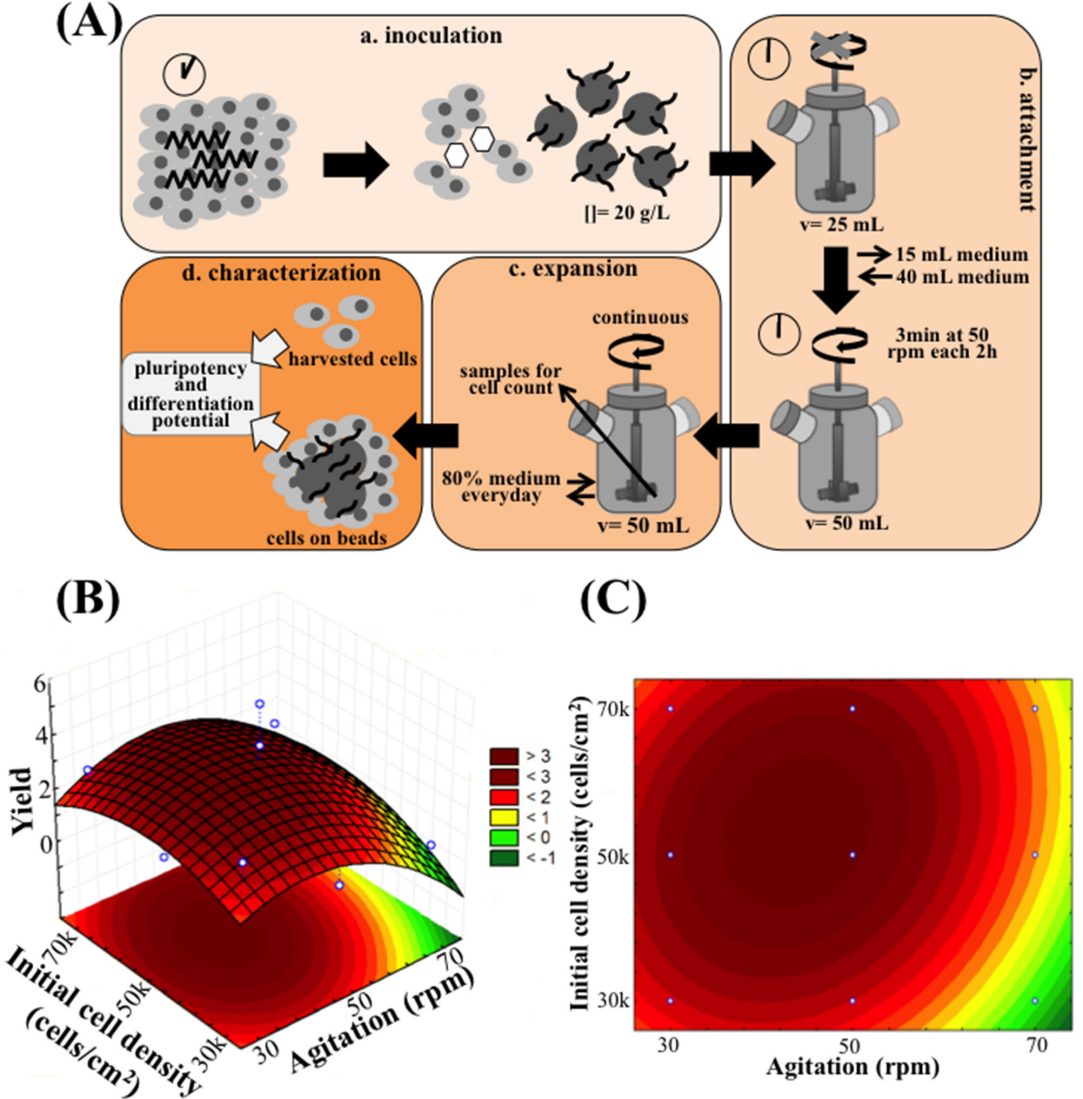
Scaling-up hiPS cell expansion in E8 medium to a 50 mL spinner flask, under dynamic conditions. (A) The procedure consists in 4 steps: a. EDTA+ROCKi inoculation strategy into 20 g/L VtnM; b. Attachment is performed in half of the total volume in static conditions for 24 h (in the presence of ROCKi), following by intermittent stirring overnight to allow cell- microcarrier interaction; c. Expansion is performed with a specific continuous agitation with 80% E8 medium change everyday; and d. At the end of the culture, cell pluripotency and differentiation potential are evaluated. Quadratic model relating initial cell density and agitation speed with cell yield during spinner flask culture of human iPS cells: 3D representation (B) and 2D heat map (C) are shown. For initial cell density: 30 000 cells/cm^2^ (−1 level) ≤ Initial Cell Density ≤ 70 000 cells/cm^2^ (1 level); for agitation speed: 30 rpm (−1 level) ≤ Agitation Speed ≤ 70 rpm (1 level).

The next step was to implement a dynamic microcarrier-based system in 50 mL-spinner flasks, envisaging the scalability of the expansion process. The protocol followed for the expansion experiments in the spinner flask is presented in Fig 3A, and is composed of four steps. On step (a) cells were inoculated in the spinner flask, using the EDTA/ROCKi method, in VtnM (20 g/L, corresponding to 360 cm^2^ of superficial area) and using half of the working volume (25 mL E8 medium supplemented with ROCK inhibitor). On Step (b), attachment of the cells to the VtnM is initiated. This step corresponds to the initial 2 days of culture. The spinner flask was operated under static conditions during the first 24 h, which is a critical period for the success of the culture that depends on cell attachment efficiency. In our experiments, attachment efficiencies of hiPS cells to VtnM were very similar, 32.5±0.9%, for inoculations with different initial cell densities. Step (b) also involved the second day of culture when ROCK inhibitor was removed from the medium, working volume was established at 50 mL and spinner flask was operated at an intermittent agitation (3 min at 50 rpm every 2 h) to maximize cell-cell and cell-microcarrier interactions. Step (c) corresponds to the period of cell expansion, which started with the initiation of the exponential growth phase at day 3 and ceased when the maximum cell yield was attained, between days 7 and 11, depending on culture conditions. During this step, the spinner flask was operated under a continuous agitation, 80% of the medium was changed everyday and samples were taken each day for cell counting. The final step (d) involved the characterization of the hiPS cells cultured on VtnM in the stirred spinner flask, through analysis of their pluripotency state and their differentiation potential by flow cytometry and immunocytochemistry.

The most critical parameters for cell expansion in the spinner flask were identified to be the initial cell density and the agitation speed, which were already evaluated for the culture of hES cells as aggregates in stirred suspension bioreactors [25]. Therefore, a face-centered composite design (FC-CD) was implemented to evaluate the influence of these two parameters on hiPS cell expansion, in terms of the maximum cell yield of the culture (S1 Table). In the factorial design, the selected values for the initial cell density were 3, 5 and 7 x10^4^ cells/cm^2^ and for the agitation speed, values were 30, 50 and 70 rpm, which were selected taking into consideration the operating conditions already reported for dynamic microcarrier cultures with hESC and mESC [12,33,34,35]. The equation that describes the quadratic model obtained for cell yield response is:

$$Yield=3.298-1.610\;X_{1}-2.408{X_{1}}^{2}+0.677\;X_{2}-1.448\;{X_{2}}^{2}+0.700\;X_{1}X_{2}$$

where *X*_*1*_ is the agitation rate and *X*_*2*_ is the initial cell density.

The second order polynomial generated for cell yield in a spinner flask culture does not fully describe the expected experimental data (*R*^2^ = 0.479), which could be anticipated due to the inherent variability of this cell culture system. Nevertheless, based on the regression model, response surface plot and 2D heat plot were established as shown in Fig 3B and 3C. The optimal conditions predicted by the model to reach a maximum cell yield of 3.5 were 55,000 cells/cm^2^ for the initial cell density and 44 rpm for the agitation rate.

### hiPS cell expansion under optimized dynamic culture conditions

**Fig 4:**
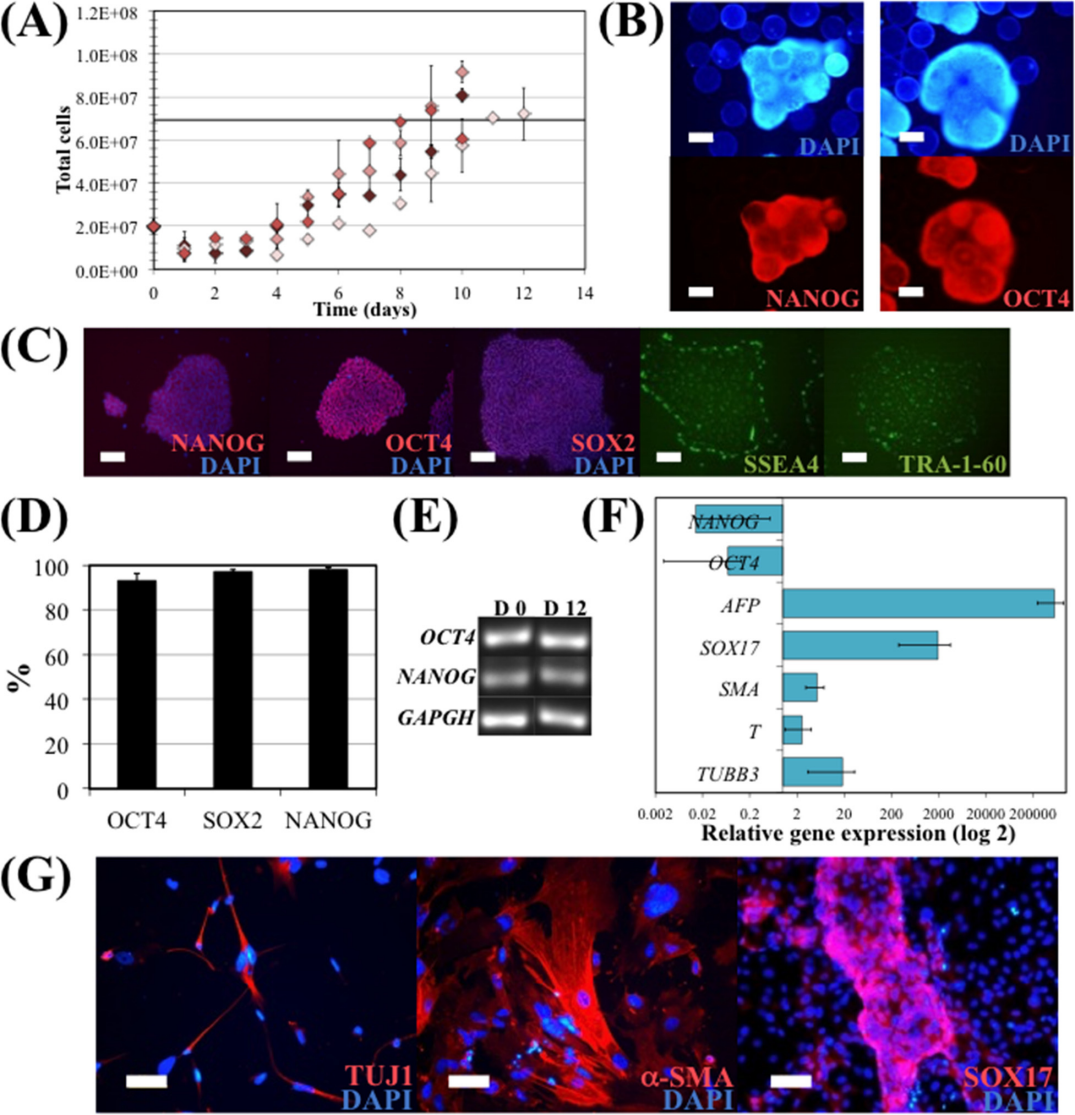
hiPS cell expansion in E8 medium, in a 50 mL spinner flask. EDTA clump inoculation was performed using 55,000 cells/cm^2^ and a continuous agitation at 44 rpm (optimal values obtained solving the quadratic model). (A) Total cell numbers during the 10 days expansion. Maximum cell yield obtained solving the quadratic model is represented by the black line. Error bars represent SEM (standard error of the mean) of duplicate samples. (B) Immunostaining of hiPS cells cultured on microcarriers in the spinner flask with E8 medium at day 10. Cells were stained for pluripotency markers NANOG (left panel) and OCT4 (right panel), and nuclei counterstained with DAPI. Scale bars– 100 μm. (C) Immunostaining of hiPS cells harvested from microcarriers after 10 days expansion in spinner flask and re-plated on GP. Cells were stained after 3 days for intracellular pluripotency markers NANOG, OCT4 and SOX2, and nuclei counterstained with DAPI; and for surface markers SSEA4 and TRA-1-60. Scale bars– 100 μm. (D) Flow cytometry analysis of hiPS cells harvested from microcarriers after 10 days expansion in spinner flask. Cells were stained for OCT4 and NANOG. (E) mRNA was isolated from hiPSC at day 0 and at the end of the spinner flask culture (day 12) on microcarriers, and the undifferentiated hiPSC marker transcripts (*OCT4* and *NANOG*) were analysed by RT-PCR. (F) Quantitative RT-PCR analysis of spontaneous differentiated EB of hiPSC cultured in spinner flask. The relative expression of each gene was measured against the same gene prior to differentiation. (G) Immunostaining showing the formation of cells expressing SOX17 (endoderm), TUJ1 (ectoderm) and α-SMA (mesoderm) after EB formation and spontaneous differentiation assay with hiPSC cultured in spinner flask. Nuclei were counterstained with DAPI. Scale bar: 50 μm.

In order to verify the validity of the proposed model, several runs of hiPS cell expansion in spinner flask were performed under the optimum conditions given by the FC-CD, which were an initial cell density of 55,000 cells/cm^2^ and an agitation rate of 44 rpm. Maximum cell numbers achieved in these experimental culture runs were compared with the model-predicted value of the maximum cell number achieved in a culture under the optimal conditions (Fig 4A). Predicted and experimental results for maximum cell yield are similar, 3.5 and 4.0±0.4, respectively. Interestingly, the values of the maximum cell yield obtained in the experimental culture runs were all above the value predicted by the model. Therefore, although the inherent variability of hiPS cell expansion on microcarriers under dynamic conditions, in a spinner flask, the model obtained by the FC-CD proved to be a useful approximation for this culture system.

hiPS cells expanded under these optimal conditions were then evaluated for their pluripotency and undifferentiated state. It was confirmed that hiPS cells growing attached onto VtnM-coated microcarriers in a spinner flask retained their pluripotency characteristics, since these cells presented NANOG and OCT4 expression, as detected by immunocytochemical analysis. Also, the cells maintained their capacity to form typical undifferentiated colonies when harvested from microcarriers and re-plated on GP, since they stained positively for the pluripotency markers NANOG, OCT4, SOX2, SSEA4 and TRA-1-60 (Fig 4C).

Pluripotency maintenance was demonstrated by flow cytometry analysis. As shown in Fig 4D, more than 93% of the cells were positive for the pluripotency markers NANOG, SOX2 and OCT4 after 12 days of culture. mRNA was isolated from hiPS cells at day 0 and at the end of the spinner flask culture (day 12) in order to assess for the expression of the hiPS cell markers *OCT4* and *NANOG*, by RT-PCR (Fig 4E). It was confirmed that cells cultured in spinner flasks maintained gene expression of the pluripotency markers. Furthermore, hiPS cells collected at the end of a spinner flask culture retained a normal karyotype (46 XX).

hiPS cell pluripotency was also evaluated in terms of their ability to differentiate into progeny of the three germ layers, which was assessed *in vitro* by induction of spontaneous differentiation of cells harvested from VtnM-coated microcarriers at the end of a spinner flask culture. Embryoid body (EB) formation was achieved using hiPS cells that were harvested, re-plated and cultured on GP, and finally inoculated in low-attachment plates to form cell aggregates in suspension. Cells were able to aggregate as EB and were cultured for 5 weeks. Quantitative reverse transcriptase-polymerase chain reaction showed upregulation of genes associated with the formation of the three germ layers: endoderm (*SOX17*, *AFP*), ectoderm *(TUBB3*) and mesoderm (*T* and *SMA*); and downregulation of pluripotency markers (*OCT4* and *NANOG*) (Fig 4F). Furthermore, expression of SOX17, TUJ1 and α-SMA markers, representing the three germ lineages; endoderm, ectoderm and mesoderm, respectively, was observed by immunostainning of the differentiated cells re-plated on laminin/poly-D-lysine-coated well plates (Fig 4G).

### Differentiation potential of hiPS cells cultured in dynamic conditions

Two different experimental settings were performed in order to evaluate the differentiation potential of hiPS cells after expansion under dynamic conditions in the spinner flask: directed cardiomyocyte (CM) differentiation and commitment to neural progenitor (NP) cells. Both directed differentiation protocols were performed by a) plating microcarriers with hiPS cells in low-attachment plates, and b) plating hiPS cells harvested from microcarriers on GP.

**Fig 5:**
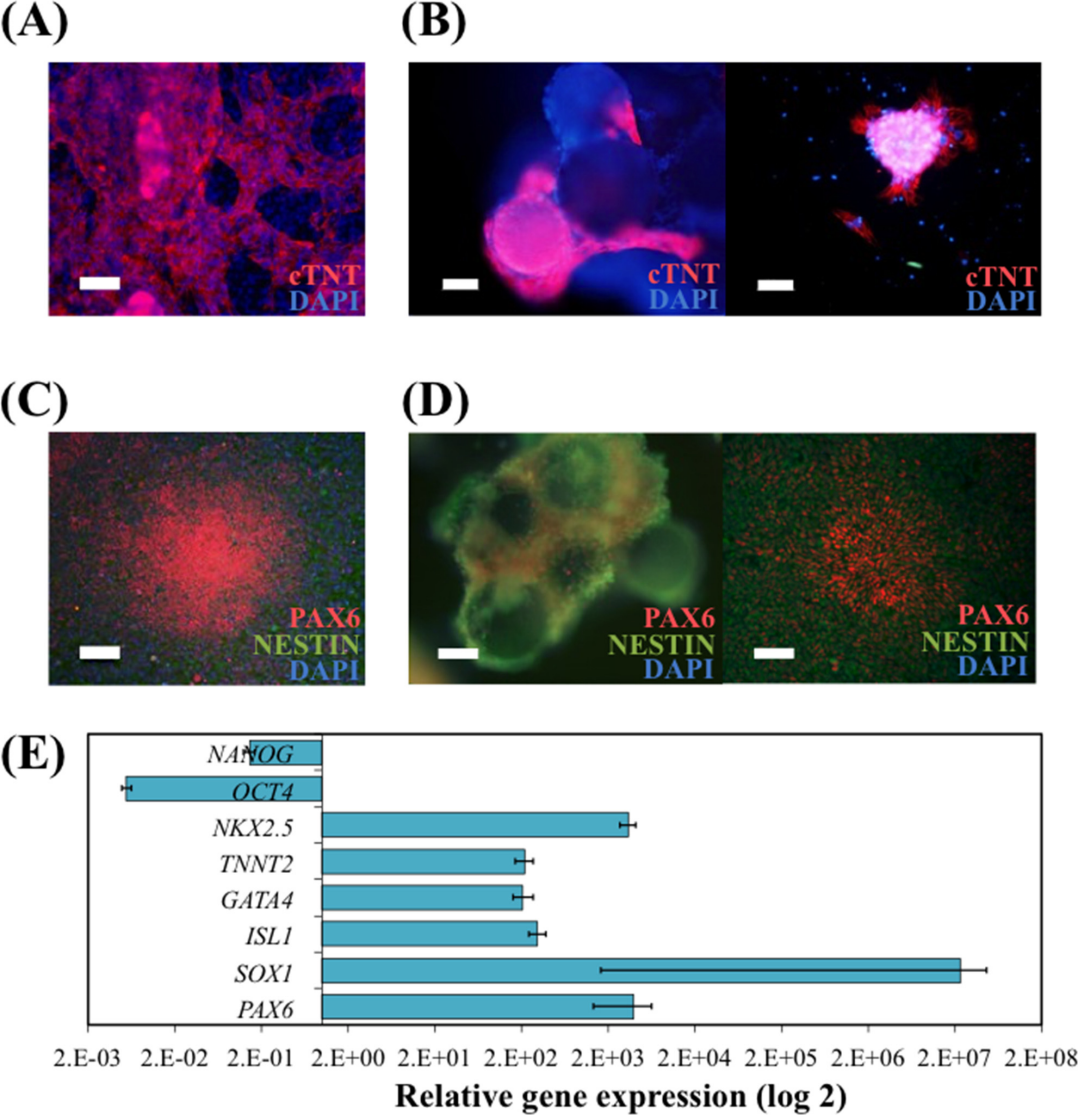
hiPS cells cultured in spinner flasks with E8 medium and VtnM retain their differentiation potential. Cardiomyocyte (CM) differentiation was performed using Life Technologies Cardiomyocyte Differentiation Kit (A) of hiPS cells harvested from microcarriers and re-plated on GP. Immunostaining depicting CM for the cTNT marker. Nuclei were counterstained with DAPI. Scale bar– 100 μm; and (B) of hiPS cells on VtnM in static conditions in a low attachment plate. Immunostaining for the cTNT marker depicting CM on microcarriers (left panel) and plated CM on GP after differentiation protocol (right panel). Nuclei were counterstained with DAPI. Scale bars– 50 μm. Neural induction by Dual-SMAD inhibition (C) of hiPS cells harvested from microcarriers and re-plated on GP. Immunostaining depicting neural progenitor (NP) cells for the neuroectoderm markers PAX6 and NESTIN. Nuclei were counterstained with DAPI. Scale-bar: 100 μm; and (D) of hiPS cells on VtnB in static conditions in a low attachment plate. Immunostaining for the neuroectoderm markers PAX6 and NESTIN depicting NP cells on microcarriers (left panel) and plated NP cells on GP after differentiation protocol (right panel). Nuclei were counterstained with DAPI. Scale bars– 50 μm. (E) Quantitative RT-PCR analysis of differentiated hiPSC cultured in spinner flask, subject to cardiomyocytes or neural differentiation. The relative expression of each gene was measured against the same gene prior to differentiation.

Directed differentiation of hiPS cells into CM was performed after the spinner flask culture. Spontaneous contracting regions in GP (S1 Video) and beating cell-VtnM aggregates (S2 Video) on low-attachment plate were observed at day 10 of differentiation. CM induction was confirmed at day 16 by immunocytochemistry analysis (Fig 5A and 5B) since cTNT^+^ cells were obtained both on GP and on VtnM. Also, upon re-plating of CM obtained on VtnM onto GP, after the differentiation protocol, it was possible to observe the presence of contracting colonies (S3 Video) that stained positively for the cardiac marker cTNT.

NP cells were also obtained from spinner flask-expanded hiPS cells by dual inhibition of SMAD signaling [36]. After a 12 day-differentiation protocol on GP and on VtnM with hiPS cells cultured in spinner flask, immunocytochemical analysis showed strong expression of the early neural differentiation markers PAX6 and NESTIN, whereas the expression of the pluripotency marker OCT4 was not observed (Fig 5C and 5D). Also, NP cells that were obtained on VtnM, were re-plated on GP after the differentiation protocol and after 4 days it was observed neuroepithelial cells arranged in neural rosette structures, which expressed PAX6 and NESTIN markers.

In Fig 5E are presented the relative gene expressions obtained by quantitative RT-PCR after cardiac and neural differentiation. It was demonstrated an increase of transcription levels of representative genes of cardiac markers (early markers *ISL1* and *GATA4* and late markers *TNNT2* and *NKX2*.*5*) or increase of transcription levels of representative genes of neural progenitor markers (*PAX6* and *SOX1*), while there was a decrease in pluripotency marker gene expression (*OCT4* and *NANOG*).

## Discussion

Biomedical applications of stem cell-derived products depend on the availability of large numbers of cells, or their differentiated progeny. However, developing GMP-compliant scalable and efficient process for stem cell production, namely hiPS cell expansion, followed by directed differentiation into progenitor cells and then fully mature cells, is still a challenge.

*In vitro* expansion of hPS cells relies on cell-ECM interaction that occurs between cell surface adhesion molecules, and enables cells to attach and proliferate. Geltrex (or Matrigel™) is an undefined mixture of ECM proteins extracted from the Engelbreth-Holm-Swarm (EHS) mouse tumors, then, its quality and composition varies from lot to lot. Also, there are safety concerns over this substrate use in clinical applications due to the risk of contamination with animal-derived pathogens and immunogens [37,38]. Multiple matrix proteins, such as laminin [39,40], vitronectin [22,41] and fibronectin [42,43], support hPS cell growth in their undifferentiated state. Vitronectin protein can be found in both serum and the ECM and mediates cell adhesion and spreading, and it is relatively easy to overexpress and purify [22], thus being a very promising xeno-free substrate to support the cost-effective scale-up of hiPS cell proliferation.

Although TeSR™ medium has been used for hPS cell expansion in the complete absence of animal proteins, the inclusion of human serum albumin (HSA) and human-sourced matrix proteins makes the production process expensive and impractical for scale-up. Recently, basic components of hES cell and iPS cell culture were re-optimized in the absence of BSA and β-mercaptoethanol (BME, a toxic component in the absence of BSA) and a completely defined medium, E8 (eight components, including DMEM/F12), was developed [19]. E8 medium reduces process cost and simplifies quality control, being a promising medium for studying specific signaling pathways in self-renewal and differentiation, due to its simple composition. Therefore, the use of E8 medium may facilitate the hiPS cell research transfer to the clinic. In the present work, it was demonstrated that Vtn-coated surface combined with E8 medium can support hiPS cell expansion and serial passaging in tissue culture plates, while cells maintain their undifferentiated and pluripotent states. In parallel, with the development of cell culture medium, a consistent xeno-free dissociation method is also important. Conventionally, hPS cells are passaged as aggregates, using and enzymatic treatment, but this process is always accompanied with an excessive cell death. It was reported recently that, after a specific EDTA treatment, hiPS cells could be partially dissociated to generate small aggregates (3 to 5 cells) that survived [26] and attached to a GP within minutes, spread in 2 h and presented a colony-like morphology in 24 h. These results were confirmed in our work since we demonstrated that our hiPS cell model retained stable proliferation and pluripotency markers after growth on Vtn surface combined with E8 medium, for four consecutive passages, using EDTA treatment.

We have also demonstrated that by coating polystyrene microcarriers with Vtn substrate, the model hiPS cell line was effectively expanded in a suspension culture in static conditions. The optimization for the inoculation protocol indicated that higher cell yields are obtained when cells are inoculated as small clumps (3–6 cells) using EDTA treatment (5 min), in the presence of ROCK inhibitor, for the first 24 h of culture. The cell yields achieved in the microcarrier suspension culture were comparable to the ones obtained when cells were cultured in tissue culture plates.

The microcarrier-based culture is an attractive system due to the scale-up potential for hPS cell expansion, when combined with stirred bioreactors. Thus, spinner flask was used in this work for scaling-up of hiPS cell culture on VtnM in E8 medium. As it was mentioned before, the process optimization is required for the development of successful cell-based therapies [24]. The rational design of experiments is an interesting technique to develop a predictive mathematical model to identify critical conditions of the bioprocess system and to understand their impact in the culture output. Using a multifactorial approach and a response surface methodology we were able to evaluate the influence of agitation rate and the initial cell density on the cell yield of the culture. Results from the two-level factorial design suggested that the agitation rate has a negative effect (-1.610) on cell yield, and on the other hand the initial cell density has a positive effect (+0.677). It also suggested that the yield response is more affected by the agitation rate parameter, which is related to the shear force effect on the cells. The second order terms of both parameters were negative, indicating a downward concavity of the model, which suggests the existence of a maximum response within the range of the analyzed values, i.e. there is an optimum value of each culture parameter. In the case of agitation rate, lower rates would result in less efficient oxygen and nutrients transfer, lower mixing and larger microcarrier aggregates, however higher rates result in higher shear stress values. The calculated maximum shear stress values for this culture system, following Nagata correlations [44], varied between 0.08 and 0.26 Pa when using agitation rates between 30 and 70 rpm. These values are well below the predicted value of 0.65 Pa at which significant effects on human embryonic kidney cell morphology occur [45] and the value of 0.78 Pa at which extensive murine embryonic stem cell damage and no proliferation were noted [46]. However, by analyzing the growth curves that correspond to the 70 rpm cultures (S1 Fig), it was interesting to notice that cell expansion only occurred when inoculating at the highest density (7x10^4^ cells/cm^2^) and still in this case the culture presented a larger lag phase. In relation to cell inoculation densities, the initial cell number will affect the maximum yield of the culture due to the lack of critical autocrine signals in low density cultures or the buildup of toxic metabolites in high densities cultures [25].

An optimal response was achieved indicating that the optimal conditions were 44 rpm for agitation rate and 55,000 cells/cm^2^ for initial cell density, which corresponded to an expected yield of 3.5 that was validated and confirmed experimentally. This means that with the culture system implemented, a maximum hiPS cell density of 1.4x10^6^ cells/ml could be obtained. Importantly, cells cultured in this system maintain their pluripotency state and presented a normal karyotype. hiPS cells harvested from microcarriers at the end of the spinner flask culture were able to differentiate into derivatives of the three embryonic germ layers through EB formation and spontaneous differentiation.

Envisioning the incorporation of both expansion and differentiation steps in an integrated bioprocess, the use of microcarrier technology to directly generate hiPS cell-derived- NP cells and CM without harvesting the cells after the expansion period was evaluated, using serum-free and xeno-free conditions. We were able to efficiently differentiate hiPS cells attached to VtnM, which were previously cultured in a stirred spinner flask, to a) NESTIN^+^ and PAX6^+^ cells after 12 days of neural commitment protocol [36] and to b) clusters of beating cells after 10 days of CM differentiation (using ready-to-use media, Life Technologies). It has been already reported the generation of NP cells using this technology [47], however the system involved the use of Matrigel™ to coat the microcarriers. Also, CM were recently generated [48] using a differentiation protocol based on modulators of the Wnt signaling, nonetheless it involves the use of murine laminin to coat the microcarriers.

## Conclusion

In conclusion, a scalable and efficient bioprocess for hiPS cell expansion using xeno-free and defined conditions was developed and optimized, in order to generate larger numbers of hiPS cells, needed for clinical, drug discovery and industrial applications. Importantly, this work paves the way towards the development of strategies for the scalable integrated expansion and directed differentiation to specific lineages, (for example, neural and cardiac) of hiPS cells under defined xeno-free conditions.

## Supporting Information

S1 Fig hiPSC (Gibco hiPSC line) expansion in E8 medium, in a 50 mL spinner flask. Growth curves in terms of total cell numbers during expansion at a continuous agitation of 70 rpm. EDTA clumps inoculation was performed using 30,000 (green), 50,000 (purple) and 70,000 (blue) cells/cm^2^.
(TIF)

S1 Table Three-level face-centered composite design (FC-CD). Coded levels and correspondent values of each variable (cell density and agitation rate) of the FC-CD and experimental values of the maximum yield of the spinner flask culture for each condition of the FC-CD.
(DOCX)

S2 Table TaqMan™ assays used for quantitative real time PCR analysis. (DOCX)

S1 Video Human iPSCs (Gibco hiPSC line) cultured in spinner flasks with E8 medium and VtnB retain their differentiation potential. Cardiomyocyte differentiation was performed using Life Technologies Cardiomyocytes Differentiation Kit by plating hiPS cells harvested from microcarriers in GP. Spontaneous contracting regions on GP were observed at day 10 of differentiation.
(MOV)

S2 Video Human iPSCs (Gibco hiPSC line) cultured in spinner flasks with E8 medium and VtnB retain their differentiation potential. Cardiomyocyte differentiation was performed using Life Technologies Cardiomyocytes Differentiation Kit by plating microcarriers with hiPS cells in low-attachment plates. Beating cell-VtnM aggregates in low-attachment plate were observed at day 10 of differentiation.
(MOV)

S3 Video Human iPSCs (Gibco hiPSC line) cultured in spinner flasks with E8 medium and VtnB retain their differentiation potential. CM, obtained on VtnM after 10 days of differentiation using Life Technologies Cardiomyocytes Differentiation Kit, were re-plated onto GP and it was observed the presence of contracting colonies.
(MOV)
