# Correction: Defined Essential 8™ Medium and Vitronectin Efficiently Support Scalable Xeno-Free Expansion of Human Induced Pluripotent Stem Cells in Stirred Microcarrier Culture Systems

**DOI:** 10.1371/journal.pone.0155296

**Published:** 2016-05-05

**Authors:** Sara M. Badenes, Tiago G. Fernandes, Cláudia S. M. Cordeiro, Shayne Boucher, David Kuninger, Mohan C. Vemuri, Maria Margarida Diogo, Joaquim M. S. Cabral

The affiliation for the seventh and eighth authors is incorrect. The correct affiliation for MM Diogo and JMS Cabral is #1 Department of Bioengineering, and Institute for Bioengineering and Biosciences, Instituto Superior Técnico, Universidade de Lisboa, Lisboa, Portugal. The affiiliations should appear as shown here: Sara M. Badenes^1^, Tiago G. Fernandes^1^, Cláudia S. M. Cordeiro^1^, Shayne Boucher^2^, David Kuninger^2^, Mohan C. Vemuri^2^, Maria Margarida Diogo^1^, Joaquim M. S. Cabral^1^
