# Supplementary figures and images for: Defined Essential 8™ Medium and Vitronectin Efficiently Support Scalable Xeno-Free Expansion of Human Induced Pluripotent Stem Cells in Stirred Microcarrier Culture Systems
Source: PLoS One. 2016 Mar 21;11(3):e0151264. doi: 10.1371/journal.pone.0151264 (PMC4801338; doi:10.1371/journal.pone.0151264)

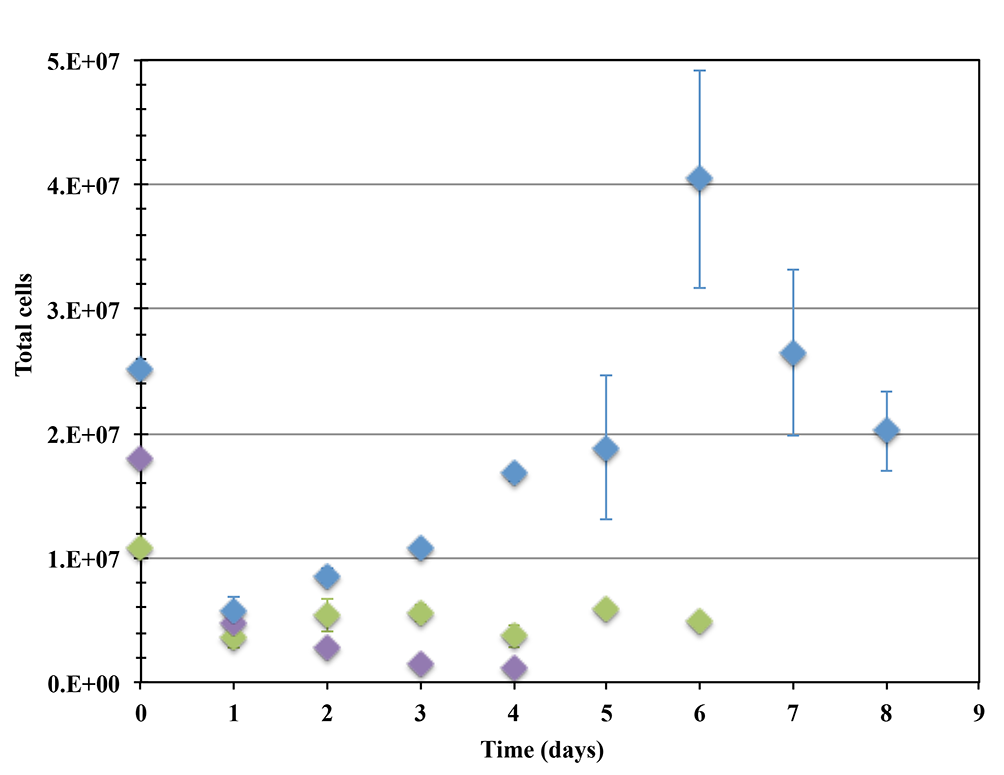

Supplement: S1 Fig — Growth curves in terms of total cell numbers during expansion at a continuous agitation of 70 rpm. EDTA clumps inoculation was performed using 30,000 (green), 50,000 (purple) and 70,000 (blue) cells/cm2. (TIF) [file pone.0151264.s001.tif]
