# Supplementary material for: Defined Essential 8™ Medium and Vitronectin Efficiently Support Scalable Xeno-Free Expansion of Human Induced Pluripotent Stem Cells in Stirred Microcarrier Culture Systems
Source: PLoS One. 2016 Mar 21;11(3):e0151264. doi: 10.1371/journal.pone.0151264 (PMC4801338; doi:10.1371/journal.pone.0151264)
Supplement: S1 Table — Coded levels and correspondent values of each variable (cell density and agitation rate) of the FC-CD and experimental values of the maximum yield of the spinner flask culture for each condition of the FC-CD. (DOCX) [file pone.0151264.s002.docx]

| **Condition** | **Cell density (code)** | **Speed (code)** | **Cell density (cells/cm^2^)** | **Agitation rate (rpm)** | **Maximum Yield** |
| --- | --- | --- | --- | --- | --- |
| 1 | -1 | -1 | 30000 | 30 | 3.11 |
| 2 | -1 | 1 | 30000 | 70 | 0.55 |
| 3 | 1 | -1 | 70000 | 30 | 2.77 |
| 4 | 1 | 1 | 70000 | 70 | 1.61 |
| 5 | -1 | 0 | 30000 | 50 | 0.64 |
| 6 | 1 | 0 | 70000 | 50 | 1.95 |
| 7 | 0 | -1 | 50000 | 30 | 1.37 |
| 8 | 0 | 1 | 50000 | 70 | 0.26 |
| 9 | 0 | 0 | 50000 | 50 | 2.98 |
| 10 | 0 | 0 | 50000 | 50 | 3.36 |
| 11 | 0 | 0 | 50000 | 50 | 5.45 |
| 12 | 0 | 0 | 50000 | 50 | 3.96 |
