# Supplementary material for: Defined Essential 8™ Medium and Vitronectin Efficiently Support Scalable Xeno-Free Expansion of Human Induced Pluripotent Stem Cells in Stirred Microcarrier Culture Systems
Source: PLoS One. 2016 Mar 21;11(3):e0151264. doi: 10.1371/journal.pone.0151264 (PMC4801338; doi:10.1371/journal.pone.0151264)
Supplement: S2 Table — (DOCX) [file pone.0151264.s003.docx]

|  | **Target gene** | **Assay ID** |
| --- | --- | --- |
| Endogeneous control | *GAPDH* | Hs02758991_g1 |
| Pluripotent | *OCT4/POU5F1* | Hs00999634_gH |
| Pluripotent | *NANOG* | Hs02387400_g1 |
| Endoderm | *SOX17* | Hs00751752_s1 |
| Endoderm | *AFP* | Hs00173490_m1 |
| Ectoderm | *b-III-tubulin/TUBB3* | Hs00801390_s1 |
| Mesoderm | *T* | Hs00610080_m1 |
| Mesoderm | *SMA4* | Hs02341953_m1 |
| Early cardiac | *ISL1* | Hs01099687_m1 |
| Early cardiac | *GATA4* | Hs00171403_m1 |
| Late cardiac | *TNNT2* | Hs00165960_m1 |
| Late cardiac | *NKX2.5* | Hs00231763_m1 |
| Neural | *PAX6* | Hs00240871_m1 |
| Neural | *SOX1* | Hs01057642_s1 |
